# A Gradient-Field Pulsed Eddy Current Probe for Evaluation of Hidden Material Degradation in Conductive Structures Based on Lift-Off Invariance

**DOI:** 10.3390/s17050943

**Published:** 2017-04-25

**Authors:** Yong Li, Haoqing Jing, Ilham Mukriz Zainal Abidin, Bei Yan

**Affiliations:** 1 State Key Laboratory for Strength and Vibration of Mechanical Structures, Shaanxi Engineering Research Center of NDT and Structural Integrity Evaluation, Xi’an Jiaotong University, Xi’an 710049, China; jinghaoqing@stu.xjtu.edu.cn (H.J.); yanbei@stu.xjtu.edu.cn (B.Y.); 2 Leading Edge NDT Technology (LENDT) Group, Malaysian Nuclear Agency, 43000 Bangi, Kajang, Selangor, Malaysia; mukriz@nuclearmalaysia.gov.my

**Keywords:** electromagnetic non-destructive evaluation, gradient-field pulsed eddy current probe, lift-off invariance, material degradation, inversion, coated conductive structures

## Abstract

Coated conductive structures are widely adopted in such engineering fields as aerospace, nuclear energy, etc. The hostile and corrosive environment leaves in-service coated conductive structures vulnerable to Hidden Material Degradation (HMD) occurring under the protection coating. It is highly demanded that HMD can be non-intrusively assessed using non-destructive evaluation techniques. In light of the advantages of Gradient-field Pulsed Eddy Current technique (GPEC) over other non-destructive evaluation methods in corrosion evaluation, in this paper the GPEC probe for quantitative evaluation of HMD is intensively investigated. Closed-form expressions of GPEC responses to HMD are formulated via analytical modeling. The Lift-off Invariance (LOI) in GPEC signals, which makes the HMD evaluation immune to the variation in thickness of the protection coating, is introduced and analyzed through simulations involving HMD with variable depths and conductivities. A fast inverse method employing magnitude and time of the LOI point in GPEC signals for simultaneously evaluating the conductivity and thickness of HMD region is proposed, and subsequently verified by finite element modeling and experiments. It has been found from the results that along with the proposed inverse method the GPEC probe is applicable to evaluation of HMD in coated conductive structures without much loss in accuracy.

## 1. Introduction

Coated conductive structures of nonmagnetic materials involving Aluminum, Copper and Titanium, etc. can be widely found in industrial fields including aerospace, nuclear, transportation, chemical engineering, etc., and normally serve in corrosive and hostile environment. Therefore, in-service coated conductive structures are usually found subject to structural corrosion whose initial exhibition is material degradation within a localized region in the structure [1]. Even though the nonmetallic protection coating is deployed over the conductor surface, coated conductive structures are still vulnerable to material degradation especially hidden under the coating. The Hidden Material Degradation (HMD) severely undermines the integrity of conductive structures, since it can barely be detected or even monitored without removing the protection coating. As a result, it is indispensable to non-invasively monitor the development of HMD in terms of the material property and thickness of degradation region before the structural failure occurs. 

As one of the advanced eddy current techniques [2,3], Gradient-field Pulsed Eddy Current technique (GPEC) has been found to be advantageous in detection and evaluation of corrosion in metallic structures in terms of high inspection sensitivity and evaluation accuracy [4]. It could be one of the promising techniques for evaluation of HMD in coated conductive structures. Whereas, two critical inspection issues must be resolved before inspection probes based on GPEC (namely GPEC probes) are applied: (1) the coating thickness may vary, which causes the variation in the distance between the probe bottom and conductor surface (namely lift-off), and thus gives rise to the lift-off noise in testing signals; and (2) intricate calculation is needed for simultaneous evaluation of the conductivity and thickness of HMD. 

Lift-off Invariance (LOI) is a phenomenon which has been discovered from previous research regarding not only harmonic eddy current testing but also transient eddy current testing. It indicates that when the lift-off varies during the inspection of a conductor, the testing signals for various lift-off scenarios intersect [5,6,7]. The intersection point (namely LOI point) parameters including the LOI amplitude (*M_loi_*, the signal magnitude at the intersection point) and LOI time (*T_loi_*, the time instant of the intersection point) are believed to be immune to the lift-off variation whilst they merely vary with specimen properties [8,9]. Giguere et al. adopted *M_loi_* of the LOI point found in pulsed eddy current testing signals for imaging of cracks in the rivets [10]. Tian et al. investigated *T_loi_* of pulsed eddy current testing signals, and utilized it for evaluation of the lift-off and metal conductivity [11]. Therefore, the first issue could be resolved by using the LOI in signals from GPEC probes. However, to the authors’ knowledge, the research of LOI regarding GPEC has been barely conducted yet. Since parallel quantification of the conductivity and thickness of HMD in coated conductive structures is a typical inverse problem of eddy current testing, inverse schemes which are mostly based on deterministic approaches and utilized for defect reconstruction could be applied to resolve the second issue. Xie et al. established a deterministic-approach-based model integrating the conjugate gradient algorithm with a finite element model for retrieving the profile of the wall-thinning defect in steam generator tubes of nuclear power plants via eddy current testing [12]. Bai et al. proposed an inverse scheme for fast crack reconstruction based on transient slices and spectral components of pulsed eddy current signals [13]. Nevertheless, few researches have been carried out for investigation and implementation of an inverse scheme based on parameters of LOI point in testing signals from GPEC probes for simultaneous evaluation of HMD parameters including the conductivity and thickness of degradation region.

In light of this, the GPEC probe for evaluation of HMD in coated planar conductors is intensively investigated in this paper. The closed-form expression of the testing signal from the GPEC probe is formulated based on the analytical modeling i.e., the Extended Truncated Region Eigenfunction Expansion [14]. The correlations of the amplitude and time instant of the LOI point (*M_loi_* and *T_loi_*) in GPEC signals with the conductivity and thickness of HMD region are investigated through theoretical study. Based on this, in conjunction with Levenberg–Marquardt algorithm [15] a fast inverse method based on LOI of GPEC for quantitative assessment of HMD is proposed. The proposed inverse method is subsequently verified via finite element modeling and experiments. 

## 2. Field Formulation of GPEC and Investigation of LOI

### 2.1. Field Formulation

**Figure 1:**
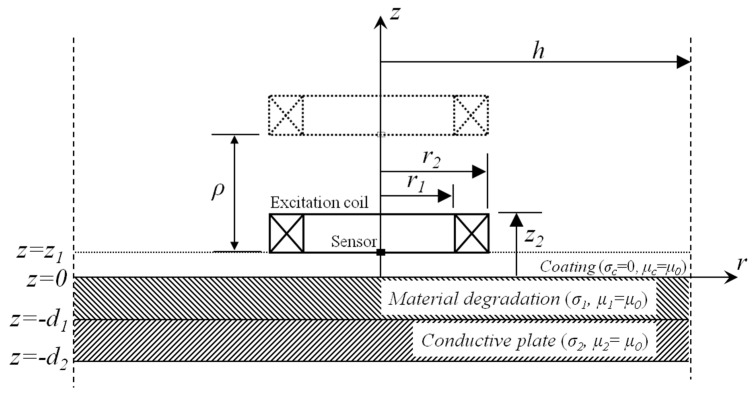
2D axi-symmetric model of a GPEC probe over a coated nonmagnetic conductor with HMD.

The structure of the GPEC probe is similar to that of pulsed eddy current testing except that magnetic sensors deployed in the excitation coil are used for measuring gradient magnetic field in lieu of absolute magnetic field. Suppose that a cylindrical GPEC probe consisting of an excitation coil for generation of incident magnetic field and magnetic sensor for sensing the net gradient field is deployed over a coated nonmagnetic conductor with HMD. The model is shown in Figure 1. It is assumed that the length and width of the HMD area are considerably larger than the outer diameter of the excitation coil. Based on the analytical modeling [14,16], the GPEC signal concerning the *z*-direction gradient field of the *z*-component of net magnetic field is formulated as:

(1) $$g_{z}\left(B_{z}\right)=\frac{\partial B_{z}}{\partial z}=\frac{\mu_{0}NI(t)}{H\left(r_{2}-r_{1}\right)h^{2}}⊗\sum_{i=1}^{\infty }J_{0}\left(a_{i}r\right)\left[e^{a_{i}z}-e^{-a_{i}(z+2\rho )}\eta_{i}(t)\right]\left[e^{-a_{i}z_{1}}-e^{-a_{i}z_{2}}\right]\frac{\chi \left(a_{i}r_{1},a_{i}r_{2}\right)}{{\left[a_{i}J_{0}\left(a_{i}h\right)\right]}^{2}}$$

where, ⊗ denotes convolution. *H* is the coil height, *H* = *z*_2_ − *z*_1_. *ρ* is the variation in the probe lift-off, *ρ* ≥ 0. *I*(*t*) and *N* are the excitation current and number of turns of the excitation coil. *μ*_0_ is the permeability of vacuum. *J*_0_ denotes the Bessel function. *a_i_* is the positive root of *J*_1_(*a_i_h*) = 0. The coil coefficient *χ*(*a_i_r*_1_, *a_i_r*_2_) can be computed by referring to the identity in [17,18]. *η_i_*(*t*) stands for the generalized temporal expression of the conductor reflection coefficient [16], and varies with HMD parameters i.e., the thickness and conductivity of HMD (*d*_1_ and *σ*_1_). 

Equation (1) facilitates the computation of transient GPEC responses to the coated nonmagnetic conductor with HMD. By referring to the previous study revealing that LOI can only be found in the first-order derivatives of the eddy current testing signals against time [11], the first-order derivatives of the testing signals from the GPEC probe against time are further formulated. The LOI point could be identified by finding the intersection between *g_z_’*(*B_z_*)|*_ρ ≠ ∞_* and *g_z_’*(*B_z_*)|*_ρ = ∞_* where:

(2) $${g_{z}^{\prime }\left(B_{z}\right)|}_{\rho \neq \infty }=\frac{\mu_{0}N\left\{\partial \left[I(t)\right]/\partial t\right\}}{H\left(r_{2}-r_{1}\right)h^{2}}⊗\sum_{i=1}^{\infty }J_{0}\left(a_{i}r\right)\left[e^{a_{i}z}-e^{-a_{i}(z+2\rho )}\eta_{i}(t)\right]\left[e^{-a_{i}z_{1}}-e^{-a_{i}z_{2}}\right]\frac{\chi \left(a_{i}r_{1},a_{i}r_{2}\right)}{{\left[a_{i}J_{0}\left(a_{i}h\right)\right]}^{2}}$$

(3) $${g_{z}^{\prime }\left(B_{z}\right)|}_{\rho =\infty }=\frac{\mu_{0}N\left\{\partial \left[I(t)\right]/\partial t\right\}}{H\left(r_{2}-r_{1}\right)h^{2}}\sum_{i=1}^{\infty }J_{0}\left(a_{i}r\right)\left[e^{a_{i}(z-z_{1})}-e^{a_{i}(z-z_{2})}\right]\frac{\chi \left(a_{i}r_{1},a_{i}r_{2}\right)}{{\left[a_{i}J_{0}\left(a_{i}h\right)\right]}^{2}}$$

*M_loi_* and *T_loi_* of the identified LOI point, which are invariant to *ρ*, can be subsequently extracted for investigation of LOI characteristics of GPEC. It is noted that GPEC probes are mostly driven by the exponential excitation current written as [19]:

(4) $$I\left(t\right)=i_{0}\left(1-e^{-\frac{t}{\tau }}\right)u(t),\;u(t)=\left\{ \begin{array}{l} 0,\;t<0\\ 1,\;t\geq 0 \end{array}\right.$$

where, *i*_0_ and *τ* denote the maximum magnitude and rising time of the excitation current, respectively whilst *u*(*t*) is Heaviside step function. For such case, Equations (2) and (3) can be individually rewritten as:

(5) $${g_{z}^{\prime }\left(B_{z}\right)|}_{\rho \neq \infty }=\frac{\mu_{0}i_{0}N}{\tau H\left(r_{2}-r_{1}\right)h^{2}}\sum_{i=1}^{\infty }J_{0}\left(a_{i}r\right)\left\{e^{a_{i}z}e^{-t/\tau }-e^{-a_{i}(z+2\rho )}\left[e^{-t/\tau }⊗\eta_{i}(t)\right]\right\}\left[e^{-a_{i}z_{1}}-e^{-a_{i}z_{2}}\right]\frac{\chi \left(a_{i}r_{1},a_{i}r_{2}\right)}{{\left[a_{i}J_{0}\left(a_{i}h\right)\right]}^{2}}$$

(6) $${g_{z}^{\prime }\left(B_{z}\right)|}_{\rho =\infty }=\frac{\mu_{0}i_{0}Ne^{-t/\tau }}{\tau H\left(r_{2}-r_{1}\right)h^{2}}\sum_{i=1}^{\infty }J_{0}\left(a_{i}r\right)e^{a_{i}z}\left(e^{-a_{i}z_{1}}-e^{-a_{i}z_{2}}\right)\frac{\chi \left(a_{i}r_{1},a_{i}r_{2}\right)}{{\left[a_{i}J_{0}\left(a_{i}h\right)\right]}^{2}}$$

### 2.2. Investigation of LOI of GPEC

**Figure 2:**
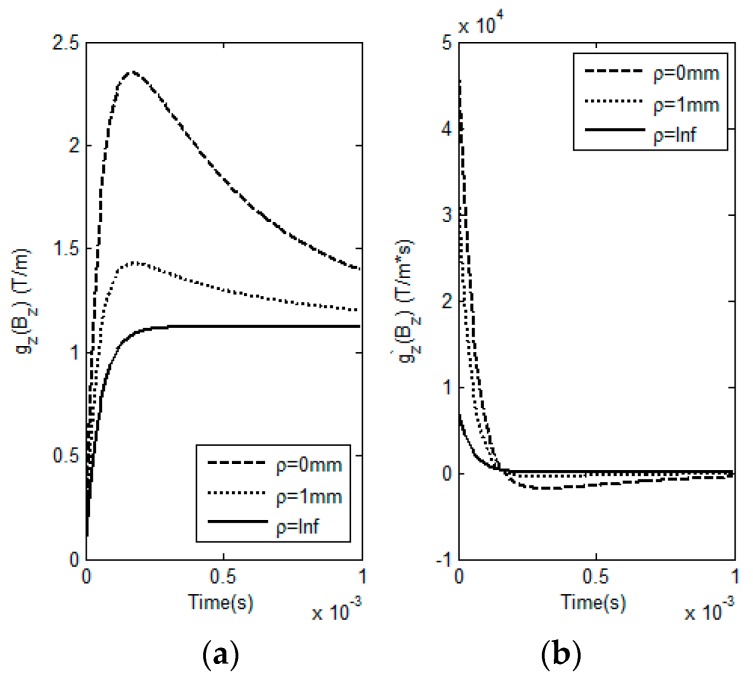
Predicted GPEC responses to the sample and their first-order derivatives against time. (**a**) GPEC signals against different lift-offs; (**b**) first-order derivatives of the GPEC signals.

A series of simulations based on Equations (1)–(6) have been conducted to analyze LOI in GPEC responses to the coated nonmagnetic conductor with variable lift-offs. The correlations of *M_loi_* and *T_loi_* of LOI points with HMD properties are investigated. The simulation parameters are listed in Table 1 and Table 2. The fundamental frequency, duty cycle and maximum amplitude of the excitation current in rectangular waveform are 100 Hz, 50% and 0.5 A, respectively. *ρ* varies from 0 mm to infinity. The computed responses from the GPEC probe to the conductor with HMD (*d*_1_ = 3 mm, *σ*_1_ = 25 MS/m) and their first-order derivatives against time are shown in Figure 2. 

**Figure 3:**
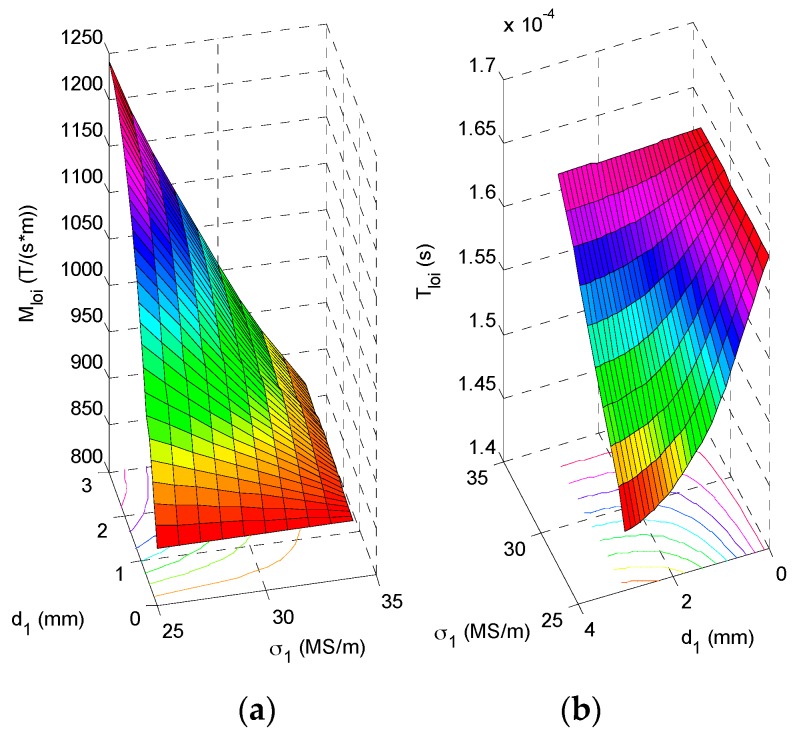
Correlations of (**a**) *M_loi_* and (**b**) *T_loi_* with *d*_1_ and *σ*_1_ of HMD.

It can be found from Figure 2 that for a HMD scenario the testing signals $g_{z}\left(B_{z}\right)$ from the GPEC probe with respect to different lift-off variations barely intersect, which implies that LOI doesn’t occur in GPEC signals. Whereas, the LOI point can be identified in $g_{z}^{\prime }\left(B_{z}\right)$. This agrees with the conclusion drawn from the work regarding pulsed eddy current testing [11]. Further investigation reveals that the LOI points of ${g_{z}^{\prime }\left(B_{z}\right)|}_{\rho \neq \infty }$ and ${g_{z}^{\prime }\left(B_{z}\right)|}_{\rho =\infty }$ nearly converge to one point, even though they vary with the lift-off. Compared with *M_loi_* and *T_loi_* of the LOI point of ${g_{z}^{\prime }\left(B_{z}\right)|}_{\rho =0}$ and ${g_{z}^{\prime }\left(B_{z}\right)|}_{\rho =\infty }$, the maximum relative errors of *M_loi_* and *T_loi_* of the LOI points of ${g_{z}^{\prime }\left(B_{z}\right)|}_{0<\rho \leq 1mm}$ and ${g_{z}^{\prime }\left(B_{z}\right)|}_{\rho =\infty }$ are less than 0.1%. This indicates that for small *ρ* the LOI points of ${g_{z}^{\prime }\left(B_{z}\right)|}_{\rho \neq \infty }$ and ${g_{z}^{\prime }\left(B_{z}\right)|}_{\rho =\infty }$ are insensitive to the variation in the coating thickness because of the nature of LOI in mitigation of lift-off influence on testing signals. Therefore, two specific lift-off cases *ρ* = 0 and *ρ* = ∞ are employed in extraction of *M_loi_* and *T_loi_* for evaluation of HMD properties. Intensive investigation regarding relations of *M_loi_* and *T_loi_* with various *d*_1_ and *σ*_1_ of HMD are subsequently conducted. During simulations, *d*_1_ varies from 0 mm to 3 mm with the interval of 0.3 mm whilst the variation in *σ*_1_ is from 25 MS/m to 34.2 MS/m with the interval of 0.9 MS/m. Different combinations of *d*_1_ and *σ*_1_ are utilized in computation of LOI parameters via Equations (5) and (6). The simulation results are exhibited in Figure 3.

It is noticeable from Figure 3 that the LOI parameters i.e., *M_loi_* and *T_loi_* are dependent of the HMD properties. When the HMD thickness is fixed, *T_loi_* is directly proportional to *σ*_1_ whilst *M_loi_* rises with *σ*_1_ decreased. In contrast, when *d*_1_ increases and *σ*_1_ is kept constant, *T_loi_* drops whilst *M_loi_* increases. This indicates that by using the LOI parameters the HMD properties could be simultaneously evaluated regardless of the variation in the coating thickness. 

## 3. LOI-Based Inverse Scheme for Evaluation of HMD Properties

Let ***u*** denote the vector of the unknown HMD properties, ***u*** = [*d*_1_, *σ*_1_]. With other parameters known and fixed, implicit expressions depicting the relations of *M_loi_* and *T_loi_* with ***u*** can be established and written as:

(7) $$M_{loi}=F_{1}(u)=F_{1}(d_{1},\sigma_{1});\;T_{loi}=F_{2}(u)=F_{2}(d_{1},\sigma_{1})$$

The functions *F*_1_ and *F*_2_ map ***u*** to the resulting amplitudes and time instants of the LOI points corresponding to different HMD properties, respectively. Suppose that ***u*** is subject to a small variation ***δ*** = [Δ*d*_1_, Δ*σ*_1_], Equation (7) is rewritten by using the Taylor series expansion as:

(8) $$\left\{ \begin{array}{l} F_{1}(u+\delta )\approx F_{1}(u)+\left[\partial F_{1}(u)/\partial u\right]\delta \\ F_{2}(u+\delta )\approx F_{2}(u)+\left[\partial F_{2}(u)/\partial u\right]\delta \end{array}\right.$$

In inversion, ***u*** is iteratively pursued until the global error function depicting the deviation of the predicted LOI parameters ***A_p_*** = [*M_loi_*, *T_loi_*] from the observation ***A_obs_*** is minimized. At each iteration step, ***δ*** is sought when minimizing the quantity $\left\|A_{\mathrm{obs}}-A_{\delta }\right\|\approx \left\|A_{\mathrm{obs}}-A_{p}-\kappa \delta \right\|$ where, ‖ . ‖denotes the norm, and ***κ*** is the 2 × 2 Jacobian matrix, ***κ*** = [∂*F*_1_(***u***)/∂***u***, ∂*F*_2_(***u***)/∂***u***]*^T^*.

**Figure 4:**
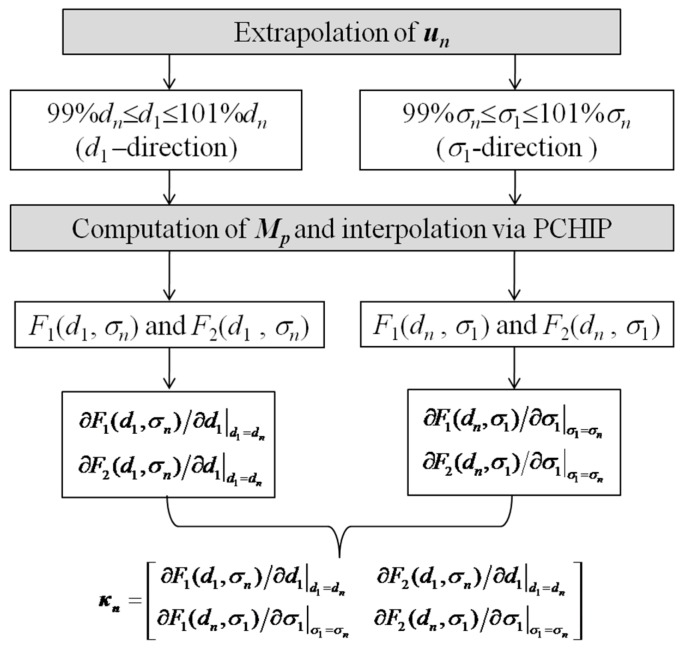
Schematic illustration of the computation of ***κ****.*

**Figure 5:**
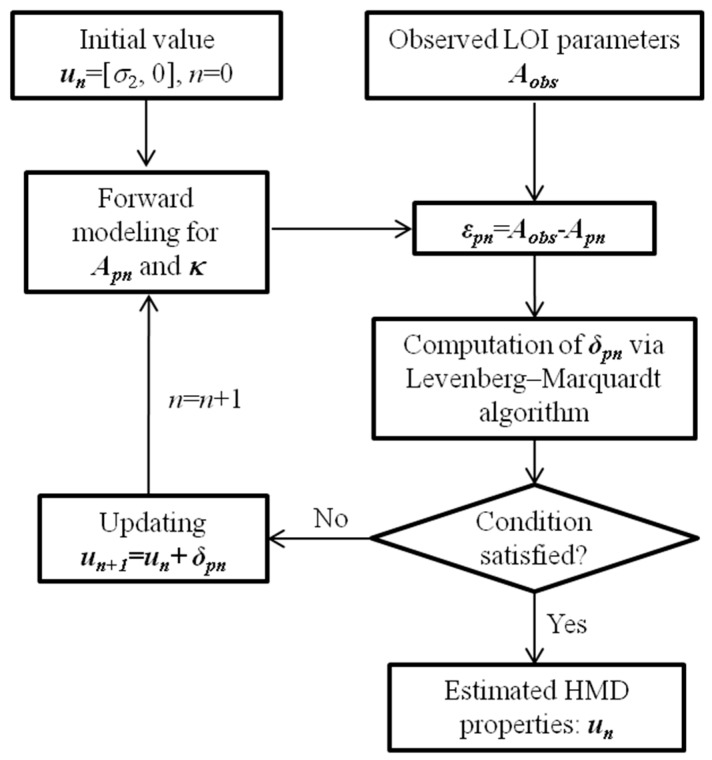
Schematic illustration of the proposed inverse scheme.

It is noted that during iteration ***A_p_*** can be readily computed by using the analytical-modeling based algorithm elaborated in Section 2.1. Compared with the computation of ***A_p_***, it is formidable to directly calculate the Jacobian matrix ***κ***. In light of this, indirect computation based on interpolation is adopted, whose schematic illustration is shown in Figure 4. At each iteration step, besides the calculation of ***A_pn_*** with an updated ***u_n_*** (*n* denoting the iteration number), ***u_n_*** is firstly extrapolated in the *d*_1_-direction (99% *d_n_*≤ *d*_1_ ≤ 101% *d_n_* whilst *σ*_1_ is fixed at *σ_n_*). The number of samples is 201. The resulting LOI parameters corresponding to defined samples are computed, and subsequently interpolated by using the Piecewise Cubic Hermite Interpolating Polynomial (PCHIP) in an effort to derive *F*_1_(*d*_1_, *σ_n_*) and *F*_2_(*d*_1_, *σ_n_*). The differentiation of *F*_1_(*d*_1_, *σ_n_*) and *F*_2_(*d*_1_, *σ_n_*) can be readily realized by using the routine ‘fnder’ in Matlab. ${\partial F_{1}(d_{1},\sigma_{n})/\partial d_{1}|}_{d_{1}=d_{n}}$ and ${\partial F_{2}(d_{1},\sigma_{n})/\partial d_{1}|}_{d_{1}=d_{n}}$ of ***κ_n_*** are thus derived from evaluation of PCHIP-based functions $\partial F_{1}(d_{1},\sigma_{n})/\partial d_{1}$ and $\partial F_{2}(d_{1},\sigma_{n})/\partial d_{1}$ with *d*_1_ = *d_n_*. The computation of ${\partial F_{1}(d_{n},\sigma_{1})/\partial \sigma_{1}|}_{\sigma_{1}=\sigma_{n}}$ and ${\partial F_{2}(d_{n},\sigma_{1})/\partial \sigma_{1}|}_{\sigma_{1}=\sigma_{n}}$ is similar to that for ${\partial F_{1}(d_{1},\sigma_{n})/\partial d_{1}|}_{d_{1}=d_{n}}$ and ${\partial F_{2}(d_{1},\sigma_{n})/\partial d_{1}|}_{d_{1}=d_{n}}$ except for the extrapolation of ***u_n_*** in the *σ*_1_-direction (99% *σ_n_*≤ *σ*_1_ ≤ 101% *σ_n_* whilst *d*_1_ is fixed at *d_n_*). With ***A_p_*** and ***κ*** computed, Levenberg–Marquardt algorithm is employed to calculate ***δ*** for updating ***u***. The LOI-based inversion terminates when the general conditions of Levenberg–Marquardt algorithm are met [18]. The schematic illustration of the proposed inverse scheme is presented in Figure 5. Note that the initial guess regarding ***u*** is referred to the HMD-free case where *σ*_1_ = *σ*_2_ and *d*_1_ = 0 mm. It should be pointed out that: (1) thanks to the analytical model utilized in inversion the proposed inverse scheme is efficient in iteratively seeking the solutions to unknown HMD properties; but (2) it can hardly be applied to evaluation of properties of the localized HMD whose size is smaller than the excitation coil in the GPEC probe. 

## 4. Corroboration via Finite Element Modeling

**Figure 6:**
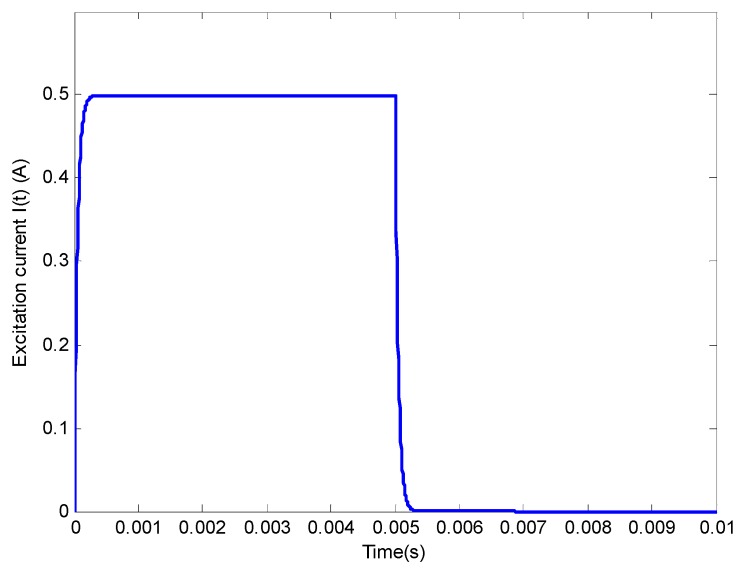
Excitation current *I*(*t*).

**Figure 7:**
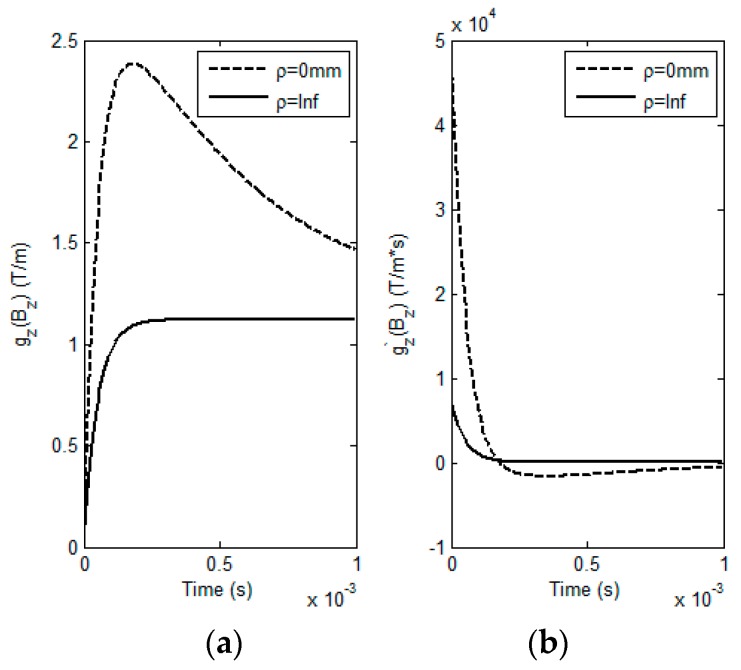
Simulated GPEC signals and their first-order derivatives against time. (**a**) GPEC signals; (**b**) first-order derivatives of the GPEC signals.

The proposed LOI-based inverse scheme is verified via finite element modeling [20,21] which gives the simulated observation corresponding to a HMD scenario with ***u*** = [1.6 mm, 28.5 MS/m]. The other model parameters are same as those listed in Table 1 and Table 2. The excitation current *I*(*t*) is exhibited in Figure 6. The acquired signals and their first-order derivatives from finite element modeling regarding GPEC are shown in Figure 7. The LOI point identified by finding the intersection point of the first-order derivative of the acquired signal with *ρ* = 0 against time and that with *ρ* = ∞. Following this, the LOI parameters are extracted and taken as the observation ***A_obs_*** = [244.4 T/m·s, 171.1 μs]. 

The observation obtained from finite element modeling and initial guess regarding ***u*** are input into the proposed inversion in an attempt to inversely retrieve the conductivity and thickness of the predefined HMD. The estimated properties of the HMD (***u_est_***) and their comparison with the true values (***u_true_***) are presented in Table 3. It is noted that in a bid to evaluate the robustness of the proposed inverse scheme, the excitation current signals with different Signal-to-Noise Ratios (SNRs) are used in finite element modeling, and thus the observed LOI parameters are polluted due to the random noise introduced to the noise-free excitation current signal. Note that: (1) the ratio of the random noise amplitude to the magnitude of the noise-free excitation current signal varies from 0% to 20%; (2) the excitation current signals with different SNRs are derived from superposition of the random noise signals and noise-free excitation current signal; and (3) *I*(*t*) used in the forward modeling regarding computation of ***A_p_*** and ***κ*** during inversion is free of noise. 

It can be seen from Table 3 that the approximated HMD properties have good agreement with the true values even though the signals are subject to noises. This implies the potential of the GPEC probe along with the LOI-based inverse scheme in simultaneous evaluation of properties of HMD in coated conductors. It is also noticeable from Table 3 that with the noise amplitude increased, the estimation accuracy of the proposed inverse scheme decreases. This indicates that before the extraction of the observed LOI parameters, proper low-pass filtering should be employed in the signal processing regarding acquired field signals in order to cancel out the extraneous noise. Further analysis of the results listed in Table 3 reveals that the accuracy in estimation of the HMD thickness is higher than that for the HMD conductivity. The reasoning could lie in the fact that the absolute value of the HMD conductivity is considerably larger than that of the HMD thickness. During inversion, the resolution of the space where the solution to an unknown HMD conductivity is sought is less than that of the solution space regarding the HMD thickness. Based on the verification via finite element modeling, the feasibility of the GPEC probe with the proposed inverse scheme in HMD evaluation is further investigated through experiments. 

## 5. Experiments

**Figure 8:**
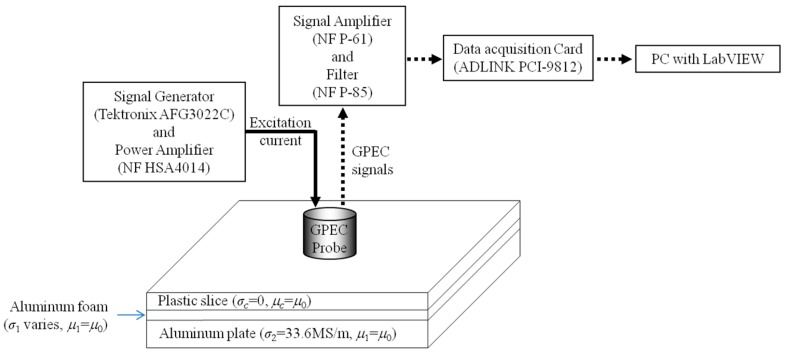
Schematic illustration of the inspection system and specimen.

A GPEC system has been built up in order to further evaluate the applicability of the GPEC probe together with the proposed inverse scheme in simultaneous assessment of properties of HMD in a coated Aluminum plate. The system setup is shown in Figure 8. The parameters of the excitation coil of the GPEC probe are same as those listed in Table 1. The pulse repetition frequency, duty cycle, maximum amplitude and rising time of the excitation current driving the excitation coil are 100 Hz, 50%, 0.3 A and 62.7 μs (measured from the acquired signal of the excitation current), respectively. A tunnel magneto-resistance sensor (MultiDimension TMR-4002, MultiDimension Technology Co., Ltd., Zhangjiagang Free Trade Zone, China) is used to sense the *z*-direction gradient field of the *z*-component of net magnetic field, and deployed at the bottom centre of the excitation coil. 

In an attempt to simulate the coated Aluminum plate with HMD, three stratified planar structures in 100 mm × 100 mm (length × width) are fabricated and taken as the specimens under evaluation. Each specimen has three layers: a plastic slice as the nonconductive protection coating; a slab of Aluminum foam as the layer of material degradation; and an Aluminum plate as the rest of the conductive region free of material degradation. Besides the specimen parameters exhibited in Figure 8, the other parameters of each specimen are listed in Table 4. It is noted the Aluminum foams with different conductivities lower than that of the Aluminum block (*σ*_2_ = 33.6 MS/m) are realized by setting different porosity densities during fabrication. Their apparent conductivities are measured by using direct current potential drop method [22,23,24,25]. A standard Aluminum sample with the conductivity and dimension of 33.6 MS/m and 300 mm × 300 mm × 5 mm (length × width × thickness), respectively is used to calibrate the system and sensor to cancel out the deviation of measured *M_loi_* and *T_loi_* against predicted values.

**Figure 9:**
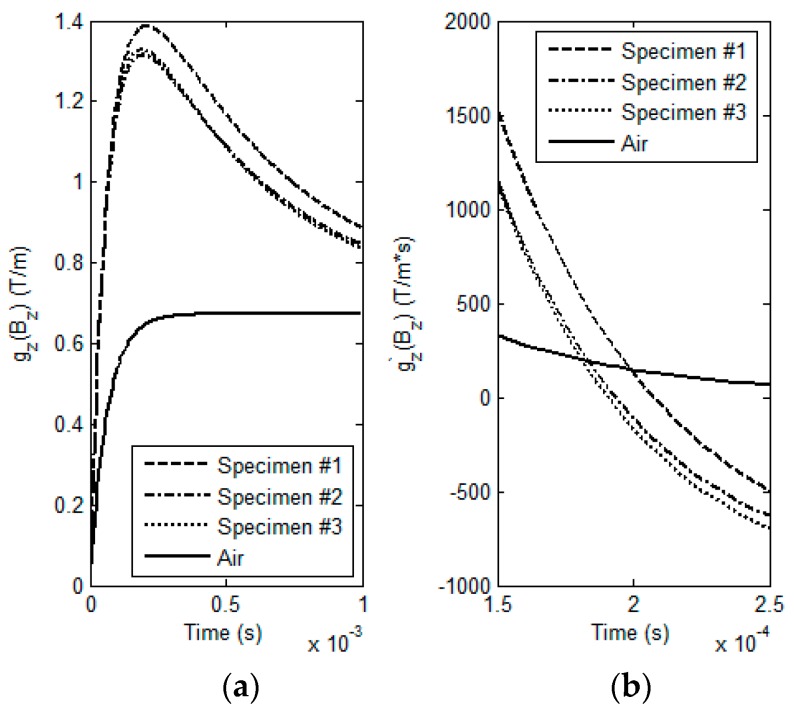
Measured GPEC signals and their first-order derivatives against time. (**a**) GPEC signals acquired in experiments; (**b**) first-order derivatives of the GPEC signals (250 μs ≥ *t* ≥ 150 μs).

Prior to the experiment with samples, the GPEC probe is firstly deployed in the air to acquire the signal of *g_z_*(*B_z_*)|*_ρ =_*_∞_. *g_z_*’(*B_z_*)|*_ρ =_*_∞_ is subsequently computed by taking the first-order derivative of *g_z_*(*B_z_*)|*_ρ =_*_∞_ against time. Following this, *g_z_*’(*B_z_*)|*_ρ_*_≠ ∞_ for each specimen is obtained via differentiation of the acquired signal of *g_z_*(*B_z_*)|*_ρ_*_≠ ∞_ against time when the probe is placed over each specimen. The experimental signals against different specimens are exhibited in Figure 9. The observed LOI parameters with respect to every specimen are subsequently acquired by extracting the amplitude and time instant corresponding to the intersection point of *g_z_*’(*B_z_*)|*_ρ_*_≠ ∞_ and *g_z_*’(*B_z_*)|*_ρ =_*_∞_: 150.3 T/m·s and 198.8 μs for Specimen #1; 192.0 T/m·s and 183.4 μs for Specimen #2; 192.0 T/m·s and 181.0 μs for Specimen #3.

The observed values are input into inversion for approximation of HMD properties of each specimen. The estimated results regarding the thickness and conductivity of simulated degradation for each specimen and their comparison with the true values are tabulated in Table 5.

It can be found from Table 5 that the estimated results have good agreement with the true values of the Aluminum foam simulating the material degradation with the relative error less than 9.0%. It is noted that thanks to the characteristics of LOI, for each specimen *M_loi_* and *T_loi_* is insensitive to thickness variation of the plastic slice. For example, regarding Specimen #1 with the coating thicknesses of 0 mm and 0.5 mm, *M_loi_* and *T_loi_* agree well with those for the original specimen setup with relative error less than 1.0%. The averaged total inversion time is 5.3 s with the number of iteration steps of 23. It can be seen from the experimental investigation that the GPEC probe in conjunction with the LOI-based inverse scheme is applicable to simultaneous evaluation of properties of HMD in coated nonmagnetic conductors. This benefits the real-time monitoring of HMD in in-service coated conductive structures. 

## 6. Concluding Remarks

In this paper, GPEC probes for evaluation of properties of material degradation hidden in coated conductive structures are intensively investigated through theoretical simulations and experiments. Closed-form expressions of GPEC responses to conductors with HMD are formulated based on the analytical modeling. A series of simulations of GPEC have been carried out for analysis of LOI characteristics and correlations between LOI parameters and HMD properties. It has been found from simulation results that LOI can only be found in the first-order derivative of the GPEC signal against time. The LOI parameters, *M_loi_* and *T_loi_* have close correlations with the HMD properties. Therefore, a fast inverse scheme based on LOI in signals from the GPEC probe is proposed for simultaneous assessment of the conductivity and thickness of HMD in planar nonmagnetic conductors. The proposed inverse method is subsequently verified by finite element modeling and experiments which give the simulated observation corresponding to the predefined HMD properties. The comparison of the estimated HMD properties and true values has revealed that the GPEC probe along with the proposed LOI-based inverse scheme is applicable for quantitatively evaluating properties of HMD in coated conductive structures without much loss in accuracy. 

In light of the technical merits of GPEC and LOI, the advantages of the proposed probe with the inverse scheme include: (1) evaluation of HMD properties regardless of small variation in the coating thickness; and (2) efficient estimation of HMD properties with high accuracy. Whereas, it should be noted that the application of the proposed technique is barely applicable to evaluation of the localized HMD whose size is smaller than the excitation coil in the probe. This opens up further work focusing on the simultaneous assessment of properties of localized HMD in coated conductive structures. 

## Figures and Tables

**Table 1:** Parameters of the excitation coil.

Coil Parameters	Value
Inner radius, *r*_1_ (mm)	11.3
Outer radius, *r*_2_ (mm)	12.5
Height, *z*_2_ − *z*_1_ (mm)	6.6
Number of turns, *N*	804

**Table 2:** Parameters of the sample.

Sample Parameters	Value
Thickness of the coating, *z*_1_ (mm)	0.64
Thickness of the degradation layer, *d*_1_ (mm)	0.0~3.0
Conductivity of the degradation layer, *σ*_1_ (MS/m)	25.0~34.2
Thickness of the conductor, *d*_2_ (mm)	5.0
Conductivity of the conductive plate, *σ*_2_ (MS/m)	34.2

**Table 3:** Comparison between approximated HMD properties and true values against different SNRs of the excitation current signals.

SNR	Inf *	26 dB	20 dB	14 dB
***u_true_***	[1.60 mm, 28.50 MS/m]			
***u_est_***	[1.63 mm, 29.05 MS/m]	[1.54 mm, 27.19 MS/m]	[1.68 mm, 26.83 MS/m]	[1.49 mm, 26.42 MS/m]
Relative error	[1.7%, 1.9%]	[3.8%, 4.6%]	[5.1%, 5.8%]	[6.9%, 7.3%]

* “Inf” denotes SNR is infinite. In such case, the excitation current signal is free of the noise.

**Table 4:** Parameters of the specimens.

	#1	#2	#3
Thickness of the plastic slice, *z*_1_ (mm)	0.2	0.5	0.2
Thickness of the Aluminum foam, *d*_1_ (mm)	0.9	1.5	1.8
Conductivity of the Aluminum foam, *σ*_1_ (MS/m)	32.3	25.9	21.2
Thickness of the Aluminum block, *d*_2_ − *d*_1_ (mm)	4.1	3.5	3.2

**Table 5:** Comparison of estimated HMD properties ***u_est_*** with the true values ***u_true_***.

	Specimen #1	Specimen #2	Specimen #3
*u_true_*	[0.9 mm, 32.3 MS/m]	[1.5 mm, 25.9 MS/m]	[1.8 mm, 21.2 MS/m]
*u_est_*	[0.86 mm, 30.63 MS/m]	[1.42 mm, 28.05 MS/m]	[1.87 mm, 19.89 MS/m]
Relative error	[4.4%, 5.2%]	[5.3%, 8.3%]	[3.9%, 6.2%]
